# Chronological changes and trend of breast cancer clinics and pathology among Iranian women during 22 years from the largest breast cancer registry in Iran

**DOI:** 10.1186/s12957-019-1757-7

**Published:** 2019-12-04

**Authors:** Sedigheh Tahmasebi, Mahsa Tajali, Majid Akrami, Peyman Arasteh, Vahid Zangouri, Aida Salehi Nobandegani, Seyed Morteza Hosseini, Afsoon Safari, Abdolrasoul Talei

**Affiliations:** 1 Breast Diseases Research Center, Shiraz University of Medical Sciences, Shiraz, Iran; 2 Non-communicable Diseases Research Center, Fasa University of Medical Sciences, Fasa, Iran; 3 Surgical Oncology Division, General Surgery Department, Shiraz University of Medical Sciences, Shiraz, Iran

**Keywords:** Clinicopathological, Breast, Cancer, Trend, Chronological change, Iran

## Abstract

**Background and objective:**

We evaluated clinicopathological changes of breast cancer (BC) during a 22-year time period among the Iranian population.

**Methods:**

This study is part of the largest BC registry in Iran. Patients were categorized as those diagnosed with BC during 1993–2005, 2006–2011, and 2012–2017 and compared regarding baseline characteristics and socioeconomical determinants, and obstetrical/gynecological and BC characteristics.

**Results:**

Overall, 688, 1871, and 3020 patients entered the 1993–2005, 2006–2012, and 2012–2017 year groups, respectively. Mean (SD) age at first presentation of BC increased throughout the year groups (47.40 ± 10.34, 49.12 ± 11.70, and 49.43 ± 12.07 years, respectively; *p* < 0.001). Mean (SD) tumor size increased from 1993–2005 to 2006–2011 and decreased onto 2012–2017 (2.82 ± 1.69, 2.91 ± 1.49, and 2.66 ± 1.52 cm, respectively; *p* < 0.001). Number of individuals with stage 4 and grade 3 BC also showed an increasing pattern (*p* < 0.001). Tumor necrosis rates showed an increase onto 2011–2017 (43%, 47.3%, and 56%, respectively; *p* < 0.001). ER positive (62.4%, 73.4%, and 77.1%, respectively; *p* < 0.001) and PR positive individuals (59.5%, 64.3%, 72.6%, respectively; *p* < 0.001) showed an increasing trend. HER2 positive expression rates increased from 1993–2005 to 2005–2011 (24.5% and 31.5%, respectively) and decreased onto 2012–2017 (31.5% and 26.8%, respectively, *p* < 0.001). Number of involved lymph nodes increased (5.70 ± 6.56, 5.65 ± 6.00, and 5.95 ± 6.99, respectively; *p* < 0.001). Pattern of BC invasion and recurrence showed significant change (*p* < 0.001).

**Conclusion:**

Clinical and pathological characteristics may be showing a changing pattern among the Iranian population.

## Introduction

Breast cancer (BC) is among the most common types of cancers among women in both developed and developing countries [1]. According to the US National Breast Cancer coalition, if a change is not implemented in the current trend of BC, on a global scale an estimated 750,000 women will die from the disease until 2030 [2]. In developing countries, BC remains to be diagnosed in late stages and this trend is showing an increase [3].

Due to changes in life style, urbanization, nutrition, and environmental factors, BCs are showing a changing pattern in the world [4]. The pattern of changes has been variable in most regions of the world depending on socioeconomic, clinical, and genetic factors [5–7]. Changes have included multiple clinical and pathological aspects of BC including esterogene (ER) receptor, progesterone (PR) receptor, and human epidermal growth receptor 2 (HER2) expression status, age of first presentation, and treatment-related characteristics [7, 8].

Recognizing the trends and patterns of change, which are mostly done based on national BC registries, facilitates correct intervention and screening programs. In the Middle East, cancer registries are scarce and little data exists on trend of changes from this region.

Inhere using data from the largest BC registry in Iran, we aimed to evaluate pattern of changes related to baseline and clinicopathological characteristics during a 22-year time period in a comprehensive manner among a large sample of the Iranian population.

## Methods and patients

### Study settings

This study is part of the largest breast cancer registry in Iran termed the Shiraz Breast Cancer Registry (SBCR). The registry is affiliated to Shiraz University of Medical Sciences and is part of the Shiraz Breast Clinic, Shiraz, Iran which is the referral center for patients with BC in Southern Iran. To date, the data base has information on baseline characteristics, social, clinical, and pathology factors from more the 8000 patients with BC. The registry has started its registration program since 2005 and includes patient diagnosed with BC since 1993; furthermore, it includes data on patients referring from multiple centers from Fars province and neighboring provinces. Protocol and study design of the registry has been described elsewhere [9].

### Patients and variables

In order to evaluate changes in BC, for this study, patients were categorized according to their time of diagnosis as followed: those diagnosed with BC during 1993 to 2005, those diagnosed during 2006 to 2011, and those diagnosed with BC during 2012 to 2017. These three groups were compared regarding baseline characteristics including age, sex, physical activity and body mass index (BMI), social determinants including job, cigarette use, alcohol use, and marital status, obstetrical and gynecological characteristics including age of menstruation, age of first pregnancy, number of abortions, number of children, number of breast feeding months, age of menopause, use of oral contraceptive medications, and use of hormone replacement therapy. Moreover, for BC specifics, the groups were assessed and compared regarding breast side involvement, age of first presentation, tumor size, number of lymph node involvements, stage, pathological grade, pathological grade of nucleus, in situ component, tumor necrosis, ER and PR receptor status, HER2 expression status, chemotherapy, invasion status, type of axillary management, type of BC operation, and site of recurrence.

### Definition of variables

Tumor size and lymph node involvement were categorized according to the TNM staging system as tumor size ≤ 2 cm, < 2 ≤ 5 cm, and > 5 cm and number of lymph nodes involved as ≤ 3, < 3 < 10, and ≥ 10 nodes involved. Staging of BC was also done according to the TNM staging system. Grade of BC, grade of nucleus, in situ component, and tumor necrosis were assessed according to pathology reports.

Regarding HER2 expression, scoring was done according to the manufacturer’s guidelines in immunohistochemistry as followed: 0 as without any staining or staining of less than 10% of cells, 1+ as weak staining in 10% of cells (staining in any part of the membrane), 2+ was considered weak to moderate staining in all of the membrane in 10% of cells, and finally 3+ was strong staining of whole membrane in 10% of cells.

Those with 0 and 1+ results were considered negative for HER2 expression. Those with 3+ results were considered positive. Those who showed 2+ (or equivocal) results using the CB11 antibody (Novocastra Laboratories, Newcastleon-Tyne, UK) had fluorescence in situ hybridization (FISH) (PathVision; Vysis, Downers Grove, IL) for evaluation of *HER2* gene amplification. Individuals with a positive FISH and a 2+ HER2 expression were considered *HER2* positive [10].

### Statistical analysis

Data analysis was performed using the Statistical Package for Social Sciences software (SPSS Inc., Chicago, Illinois, USA), software for windows® version 20. Patients were categorized into three groups based on time of diagnosis of BC. Considering that the population size diagnosed with BC before 2005 who were registered in our study was low, we categorized patients into three year groups of those diagnosed during 1993–2005, 2006–2011, and 2012–2017. Quantitative data were compared between groups using the one-way ANOVA test. For between group comparisons, the post-hoc test (Tukey post-hoc) was utilized. Qualitative data were compared between groups using the chi-square test. Data are presented as frequency and percentage or means and standard deviations (SD), where appropriate.

A *p* value of less than 0.05 was considered statistically significant.

## Results

In total, 688 patients were registered with BC during 1993–2005, 1871 individuals during 2006–2011, and 3020 individuals were registered during 2012–2017.

With regard to baseline and socioeconomic characteristics, patients with BC registered during the three year groups were significantly different regarding education status, job, marital status, duration of breast feeding, use of oral contraceptive use (OCP), and physical activity. Regarding education status, number of illiterate individuals had a steady decrease during the study year groups (24.9%, 18%, and 16.2%, respectively). Regarding job and marital status, rate of retired individuals decreased (19.2%, 14.6%, and 8.3%, respectively) and rate of married women increased (73.9%, 79.3%, and 79.9%, respectively). During the study period, duration of breast feeding (68.03 ± 52.11, 59.12 ± 42.01 and 57.55 ± 41.93 weeks, respectively) and rate of OCP use showed a decrease (60.2%, 55.1%, and 51.8%, respectively) (Table 1).

**Table 1:** Comparison of patients’ baseline characteristics between the three year groups

Variables		Year diagnosis group			*p* value
		1993–2005 (*n* = 688)	2006–2011 (*n* = 1871)	2012–2017 (*n* = 3020)	
Sex—no. (%)	Male	3 (0.4)	18 (1.0)	19 (0.6)	0.18
	Female	685 (99.6)	1853 (99.0)	3001 (99.3)	
Education—no. (%)	Illiterate	48 (24.9)^a^	117 (18.0)^b^	306 (16.2)^b^	0.01
	Primary school	53 (27.5)^a^	203 (31.3)^a^	529 (28.0)^a^	
	High school	65 (33.7)^a^	201 (31.0)^a^	647 (34.2)^a^	
	Collage education	27 (14.0)^a^	128 (22.7)^a, b^	408 (21.6)^b^	
Job—no. (%)	Retired	40 (19.2)^a^	107 (14.6)^a^	185 (8.3)^b^	0.01
	Stay at home	157 (75.5)^a^	563 (76.9)^a^	1811 (81.1)^b^	
	Governmental employee	11 (5.3)^a^	62 (8.5)^a, b^	237 (10.6)^b^	
Marital status—no. (%)	Divorced	7 (3.3)^a^	15 (2.0)^a^	38 (1.7)^a^	0.01
	Married	156 (73.9)^a^	587 (79.3)^a, b^	1840 (79.9)^b^	
	Single	48 (22.8)^a^	138 (18.7)^b^	414 (18.4)^b^	
BMI—kg/m^2^		28.06 ± 4.99	27.60 ± 4.52	27.92 ± 4.90	0.225
Age of start menstruation—yrs		13.28 ± 1.41	13.39 ± 1.92	13.26 ± 1.94	0.250
Age of first pregnancy—yrs		20.79 ± 5.46	21.50 ± 6.83	21.63 ± 7.52	0.290
No. of abortions		1.52 ± 0.86	1.51 ± 0.88	1.57 ± 1.03	0.669
No. of children		4.02 ± 1.99^a^	3.55 ± 1.77^b^	3.36 ± 1.88^b^	<0.001
Duration of breast feeding—wks		68.03 ± 52.11^a^	59.12 ± 42.01^b^	57.55 ± 41.93^b^	0.006
Age of menopause—yrs		47.31 ± 4.73	47.76 ± 6.10	48.06 ± 5.41	0.208
Use of OCP—no. (%)	Yes	127 (60.2)^a^	406 (55.1)^a, b^	1189 (51.8)^b^	0.031
	No	84 (39.8)^a^	331 (44.9)^a, b^	1105 (48.2)^b^	
Use of HRT—no. (%)	Yes	4 (1.9)	9 (1.3)	36 (1.6)	0.72
	No	204 (98.1)	708 (98.7)	2212 (98.4)	
Cigarette use—no. (%)	Yes	1 (0.5)	4 (0.5)	26 (1.1)	0.278
	No	207 (99.5)	739 (99.5)	2290 (98.9)	
Waterpipe use—no. (%)	Yes	19 (9.1)	58 (7.8)	213 (9.2)	0.492
	No	190 (90.9)	684 (92.2)	2091 (90.8)	
Use of alcohol—no. (%)	Yes	0	1 (0.1)	3 (0.1)	0.872
	No	207 (100)	739 (99.9)	2302 (99.9)	
Physical activity—no. (%)	Yes	64 (30.8)^a^	310 (41.9)^b^	932 (40.3)^b^	0.01
	No	144 (69.2)^a^	430 (58.1)^b^	1379 (59.7)^b^	

*BMI*, body mass index; *OCP*, oral contraceptive pill; *HRT*, hormone replacement therapy

All plus-minus values are means and standard deviation, unless stated otherwise. Superscript alphabets represent the results of the post-hoc test, and accordingly different alphabets show significant difference between groups. “a” is statistically different from “b” and “c”, “b” is statistically different from “a” and “c”, and “c” is statistically different from “a” and “b”

Regarding BC specifics, from 1993 to 2017 mean age at first presentation of BC showed a significant increase (47.40 ± 10.34, 49.12 ± 11.70, and 49.43 ± 12.07 years, respectively for the three year groups; *p* < 0.001). Mean tumor size showed an increase from 1993–2005 to 2006–2011 and a significant decrease onto 2012–2017 (2.82 ± 1.69, 2.91 ± 1.49, and 2.66 ± 1.52 cm, respectively; *p* < 0.001). Stage of BC showed a significant change (*p* < 0.001). Number of individuals with stage 4 BC showed a significant increase from 1993–2005 to 2006–2011 and 2012–2017 (0.4%, 1.1%, and 2.2%, respectively; *p* < 0.001).

Percentage of individuals with pathologic grade 3 BC showed a significant increase from 1993–2005 to 2012–2017 (11.9%, 18.3%, and 23.5%, respectively; *p* < 0.001); moreover, the rate of grade 3 nucleus also showed a significant increase throughout the years (8.6%, 17.1%, and 43.9%, respectively; *p* < 0.001). The rate of individuals with tumor necrosis in histological evaluation showed a significant increase onto 2011–2017 (43%, 47.3%, and 56%, respectively; *p* < 0.001). The number of ER positive individuals (62.4%, 73.4%, and 77.1%, respectively; *p* < 0.001) and the number of PR positive individuals (59.5%, 64.3%, 72.6%, respectively; *p* < 0.001) also showed a significant increase throughout the study year groups.

Regarding number of individuals with a positive HER2 expression status, an increase was seen from 1993–2005 to 2006–2011 (24.5% and 31.5%, respectively), after which a decrease was seen onto 2012–2017 (31.5% and 26.8%, respectively, *p* < 0.001).

The rate of individuals receiving chemotherapy showed a significant decrease onto 2012–2017 compared with 1993–2005 and 2006–2011 (16.1% vs. 23.1% and 23.6%, respectively; *p* < 0.001). The pattern of BC invasion showed a change during the study years. The rate of individuals with isolated vascular and lymphatic invasions, combined vascular and perineural invasion, and combined lymphatic and perineural invasion showed a decrease; however, the rate of patients with isolated perineural invasion, combined vascular, lymphatic, and perineural, combined lymphatic and vascular invasion, and those without invasion showed an increase (*p* < 0.001). Regarding the type of axillary management, comparing 2006–2011 and 2012–2017 showed that axillary node dissection (AND) rates decreased, and on the other hand, isolated sentinel lymph node biopsy (SLNB) and combined AND and SLNB showed a significant increase. Results showed that the number of involved lymph nodes in AND and SLNB showed a significant increase throughout the study period (5.70 ± 6.56, 5.65 ± 6.00, and 5.95 ± 6.99, respectively; *p* < 0.001).

Mastectomy rates also showed a significant decrease throughout the study period (71.8, 61.8%, and 36.7%, respectively).

The site of recurrence of BC showed a changing pattern. Bone metastasis increased; however, metastasis to two organs decreased (Table 2).

**Table 2:** Comparison of clinicopathological variables between the year groups

Variables	Year diagnosis group				*p* value
		1993–2005 (*n* = 690)	2006–2011 (*n* = 1871)	2012–2017 (*n* = 3020)	
Breast side involvement—no. (%)	Right	336 (50.1)	885 (48.0)	1456 (48.4)	0.62
	Left	334 (49.9)	960 (52.0)	1555 (51.6)	
Age of first presentation—yrs		47.40 ± 10.34^a^	49.12 ± 11.70^b^	49.43 ± 12.07^b^	< 0.001
Tumor size—cm		2.82 ± 1.69^a^	2.91 ± 1.49^a^	2.66 ± 1.52^b^	< 0.001
T—no. (%)†	≤ 2	376 (54.7)^a^	842 (45.0)^b^	1576 (52.2)^a^	< 0.001
	< 2 ≤ 5	283 (41.1)^a^	931 (49.8)^b^	1327 (43.9)^a^	
	> 5	29 (4.2)^a, b^	98 (5.2)^b^	118 (3.9)^a^	
N—no. (%)†	≤ 3	532 (77.4)	1419 (76.3)	2319 (76.9)	0.75
	<3 < 10	103 (15.0)	269 (14.5)	432 (14.3)	
	≥ 10	52 (7.6)	172 (9.2)	265 (8.8)	
Stage—no. (%)^†^	0	16 (3.0)^a^	51 (3.2)^a^	88 (3.3)^a^	< 0.001
	1	126 (24.0)^a^	300 (19.0)^b^	653 (24.8)^a^	
	2	254 (48.4)^a, b^	787 (49.7)^b^	1185 (45.1)^a^	
	3	127 (24.2)^a^	426 (26.9)^a^	646 (24.6)^a^	
	4	2 (0.4)^a^	18 (1.1)^a^	58 (2.2)^b^	
Grade—no. (%)	1	133 (29.9)^a^	372 (25.5)^a^	433 (17.8)^b^	< 0.001
	2	255 (57.3)^a^	817 (56.1)^a^	1399 (57.4)^a^	
	3	53 (11.9)^a^	266 (18.3)^b^	573 (23.5)^c^	
Grade of nucleus—no. (%)	1	34 (36.6)^a^	39 (25.7)^a^	195 (14.1)^b^	< 0.001
	2	51 (54.8)^a^	87 (57.2)^a^	580 (42)^b^	
	3	8 (8.6)^a^	26 (17.1)^a^	606 (43.9)^b^	
In situ component—no. (%)	Yes	194 (66.9)	1049 (69.6)	1646 (68.2)	0.516
	No	96 (33.1)	458 (30.4)	769 (31.8)	
Tumor necrosis—no. (%)	Yes	126 (43)^a^	697 (47.3)^a^	1572 (56)^b^	< 0.001
	No	167 (57)^a^	776 (52.7)^a^	940 (37.4)^b^	
Estrogen receptor—no. (%)	Positive	394 (62.4)^a^	1328 (73.4)^b^	2181 (77.1)^c^	< 0.001
	Negative	236 (37.4)^a^	475 (26.3)^b^	643 (22.7)^c^	
	Unknown	1 (0.2)^a^	6 (0.3)^a^	4 (0.1)^a^	
Progesterone receptor—no. (%)	Positive	373 (59.5)^a^	1160 (64.3)^b^	1047 (72.6)^c^	< 0.001
	Negative	253 (40.4)^a^	636 (35.3)^b^	768 (27.2)^c^	
	Unknown	1 (0.2)^a, b^	8 (0.4)^b^	4 (0.1)^a^	
HER2—no. (%)	Positive	12 (24.4)^a, b^	433 (31.5)^b^	695 (26.8)^a^	< 0.001
	Negative	22 (44.8)^a^	942 (68.5)^b^	1894 (73)^c^	
	Unknown	15 (30.6)^a^	0^b^	5 (0.2)^b^	
Chemotherapy before surgery—no. (%)	Yes	30 (23.1)^a^	106 (23.6)^a^	285 (16.1)^b^	< 0.001
	No	100 (76.9)^a^	344 (76.4)^a^	1485 (83.9)^b^	
Invasion type—no. (%)	Vascular	33 (6.1)^a^	39 (2.3)^b^	24 (0.9)^c^	
	Perineural	24 (4.4)^a^	90 (5.4)^a^	265 (9.9)^b^	< 0.001
	Lymphatic	124 (22.8)^a^	368 (22)^a^	165 (6.2)^b^	
	All	40 (7.4)^a^	135 (8.1)^a^	628 (23.6)^b^	
	None	208 (38.2)^a, b^	699 (41.8)^b^	929 (34.8)^a^	
	Vascular and perineural	14 (2.6)^a^	13 (0.8)^b^	18 (0.7)^b^	
	Lymphatic and vascular	67 (12.3)^a^	262 (15.7)^a^	600 (22.5)^b^	
	Lymphatic and perineural	34 (6.2)^a^	65 (3.9)^b^	37 (1.4)^c^	
Axillary management—no. (%)	AND	618 (99.7)^a^	1400 (78.7)^b^	1536 (53.7)^c^	< 0.001
	SLNB	2 (0.3)^a^	237 (13.3)^b^	930 (32.5)^c^	
	AND & SLNB	0^a^	143 (8)^b^	394 (13.8)^c^	
Total no. of involved lymph nodes in dissection		5.70 ± 6.56	5.65 ± 6.00	5.95 ± 6.99	< 0.001
Type of operation—no. (%)	Mastectomy	477 (71.8)^a^	1138 (61.8)^b^	1099 (36.7)^c^	< 0.001
	BCS	187 (28.2)^a^	704 (38.2)^b^	1893 (63.3)^c^	
Site of recurrence—no. (%)	Bone	43 (40.2)^b^	121 (36.4)^b^	111 (45.5)^a^	0.015
	Brain	7 (6.5)^a^	21 (6.3)^a^	18 (7.4)^a^	
	lung	16 (15)^a^	60 (18.1)^a^	36 (14.8)^a^	
	Liver	8 (7.5)^a^	56 (16.9)^b^	30 (12.3)^a, b^	
	Two organs	26 (24.3)^a^	55 (16.6)^a^	25 (10.2)^b^	
	Multiple organs	3 (2.8)^a^	9 (2.7)^a^	7 (2.9)^a^	
	Other organs	4 (3.7)^a,b^	10 (3)^b^	17 (7)^a^	

*HER*, human epidermal growth receptor; *AND*, axillary node dissection; SLNB, sentinel lymph node biopsy; *BCS*, breast conserving surgery

All plus-minus values are means and standard deviation, unless stated otherwise. Superscript alphabets represent the results of the post-hoc test, and accordingly, different alphabets show significant difference between groups. “a” is statistically different from “b” and “c”, “b” is statistically different from “a” and “c”, and “c” is statistically different from “a” and “b”

^†^*T*, *N*, and stage were calculated according to the TNM staging system

## Discussion

Inhere we evaluated trend of changes in BC clinicopathology during a 22-year period in Iran and found that a significant change regarding age of first presentation, tumor size, stage, grade, grade of nucleus, tumor necrosis, ER, PR and HER2 status, invasion status, type of axillary management, rate of chemotherapy, rate of lymph node involvement, type of operation, and pattern of recurrence was seen among patients with BC during the study period (*p* < 0.05). To the best of the authors’ knowledge, this is the largest and most comprehensive study in our region to have evaluated changes in clinicopathology of BC during a study period of more than 20 years.

In one study of the Iranian population [11] which was conducted during a 15-year period from 1986 to 2000 among five hospitals in Tehran, Iran, authors evaluated 1612 medical records among patients with BC. They found that the overall stage of BC’s and size of tumor decreased during the study period. Moreover, they found percentage of individuals with stage 2 BC to be increasing while those with stage 3 cancers to be decreasing. Aside to tumor stage which decreased in their study, the age of first presentation was unchanged during the study period. BC showed a different pattern in our study, as we found age of first presentation of BC to show an increase from 1993–2005 to 2006–2011, although similar to the mentioned study, we did find age of first presentation to be unchanged from 2006–2011 to 2012–2017. Moreover, in our study, tumor size increased during 1993–2005 to 2006–2011; however, similar to the mentioned study, we did find a decrease in mean tumor size onto 2012–2017. Furthermore, in our study rate of individuals with grade 3 tumor, stage 4 BC and tumor necrosis increased. The two studies were conducted almost 15 years apart, and our study included a very diverse group of individuals as our center is the main referral center for southern Iran and included different ethnic groups. Moreover, our registry included prospective data from patients who are routinely visited which provides more reliable findings.

One interesting finding in our study was that age of menarche remained unchanged; however, some tumor characteristics showed a worsening pattern. This shows that other factors except menstrual risk factors [12] are among the causes for which BC clinicopathology are undergoing changes.

In one study by Shankar et al. [13] conducted in India, among a total of 532 patient with BC during 1997 to 2006, they found that age of first presentation of BC had decreased throughout the study period (from 48.6 years old to 44.7 years old, *p* = 0.046). They also found that the pattern of receptor expression had changed (those with either ER or PR positive had decreased from 81.8% to 70.5%, *p* = 0.007). Moreover, they found pattern of surgical approach to BC to have changed during their study period (*p* < 0.001) as those with no surgery increased significantly from 2.3% to 26.2% from 1997 to 2006. They did not find stage of BC to show a changing pattern during the study period. In our population, unlike the mentioned study, during the 22-year period, we found age of first presentation of BC to increase. Another difference relates to status of receptor expression; our study unlike the mentioned study, showed an increasing trend in number of individuals with positive ER and PR expression.

In a report among Korean patients with BC, Seung Sang [7], using data from the national cancer registry, found that during a 10-year period from 1996 to 2006, mean age at first presentation of BC increased from 47.0 ± 10.8 to 49.2 ± 10.4 years old (*p* < 0.001), which was very similar to that of our study (47.40 ± 10.34 to 49.43 ± 12.07 years old). They also found rate of breast conserving surgery to be increasing (161% increase, *p* < 0.001), higher stages of BC (II–IV) to be decreasing (31.1% decrease, *p* < 0.001), and rates of ductal carcinoma to be decreasing as well (5.7% decrease, *p* = 0.010). Our patients also showed a significant change in overall stage of BC (an increase was seen in stage 4 BCs (0.4% to 2.2%)); moreover, they also showed a significant increase in grade 3 BCs and tumor necrosis.

Our findings are alarming as they show that although the age of first presentation of BC’s have been increasing, rates of higher grades and stages of BC have been increasing. Early screening has shown to be effective in early detection of BC at lower stages [14]. Unfortunately in Iran, despite having a lower age of first presentation for BCs compared with other regions of the world [15], screening programs have not been modified accordingly. Moreover, routine mammography screening of women is not yet generally applied in the country. Another factor which may be contributing to the changing pattern of BC’s may be related to environmental carcinogens and life style changes including tendency towards a western life style, changes in socioeconomic determinants (job, education, marital status), and changes in obstetrical and gynecological factors such OCP use, breast feeding, etc.

Another factor to be considered when evaluating changes in BC, relates to treatment protocols which have undergone changes throughout recent years. SLNB was started from 15 years ago in our institutions, breast conserving surgeries have been started in our centers from the year 2000, and most recently, intraoperative radiation therapy has been started in the past 5 years in our center.

Increased ER and PR receptor positive rates in laboratory reports may be attributed to multiple factors; these include better preservation and fixation of specimen, better antigen preservation, usage of antibodies with more affinity resulting in stronger immunohistochemical reaction throughout recent years.

We found a changing pattern in BC recurrence, invasion status, and molecular expression (ER, PR, and HER2). Although the exact etiology of this change is not known, more importantly, this shows a necessity to reconsider management and work-up plans for our population specifically. Health policies and national and local guidelines would further benefit from the results of the current report.

This study was not without limitation. The study did not include data from a national registry, and as we indicated in our protocol of study [9], our registry is a surgical oncology registry and only includes patients who have undergone surgery for BC in our centers. Consequently, patients with less aggressive forms of breast tumors have not been registered with our center. On the other hand, our population did represent diverse ethnic groups and is more representative of the Iranian population compared with other studies in the literature from our region. The changes reported regarding axillary management merely represent the introduction of SLNB to our organization, as this axillary management was introduced and applied since 2003. Prior to 2005 (the year during which official registration began), registration of patients’ data was done retrospectively, and although patients who had missing data were excluded from the current study, it should be considered that this part of the data was retrospectively collected. However, this does not compromise the main goal of the study which was to evaluate overall clinicopathological changes of BC throughout the years. We divided our study duration into three year groups of 1993–2005, 2006–2011, and 2012–2017, and considering that the majority of our patients were from more recent years, we included a wider year-group for those registered with our center before 2005.

## Conclusion

Clinical and pathological characteristics may be showing a changing pattern among the Iranian population.

## Data Availability

Data of the current study is part of a large breast cancer registry data base. Suggestion and opinions of all respectable readers and researchers are welcomed for the enhancement of future research. Readers and institutions are requested to submit their suggestions and research proposals to the Shiraz Breast Cancer Research Center at bdrc@sums.ac.ir or akramimd@yahoo.com.
